# Bactericidal Activity of Multilayered Hybrid Structures Comprising Titania Nanoparticles and CdSe Quantum Dots under Visible Light

**DOI:** 10.3390/nano11123331

**Published:** 2021-12-08

**Authors:** Ekaterina Kolesova, Anastasia Bulgakova, Vladimir Maslov, Andrei Veniaminov, Aliaksei Dubavik, Yurii Gun’ko, Olga Efremenkova, Vladimir Oleinikov, Anna Orlova

**Affiliations:** 1 School of Photonics, ITMO University, 197101 St. Petersburg, Russia; anastasiya.makovectkaya@mail.ru (A.B.); maslov04@bk.ru (V.M.); avveniaminov@itmo.ru (A.V.); adubavik@yandex.ru (A.D.); ovefr@yandex.ru (O.E.); voleinik@mail.ru (V.O.); 2 School of Chemistry, Trinity College Dublin, D02 PN40 Dublin, Ireland; igounko@tcd.ie; 3 Department of Microbiology, FSBI Gause Institute of New Antibiotics, 119021 Moscow, Russia; 4 Department of Biomaterials and Bionanotechnology, Shemyakin-Ovchinnikov Institute of Bioorganic Chemistry, 117997 Moscow, Russia

**Keywords:** hybrid nanostructures, quantum dots, photo-induced electron transfer, reactive oxygen species, antibacterial activity

## Abstract

Titania nanoparticle/CdSe quantum dot hybrid structures are a promising bactericidal coating that exhibits a pronounced effect against light-sensitive bacteria. Here, we report the results of a comprehensive study of the photophysical properties and bactericidal functionality of these hybrid structures on various bacterial strains. We found that our structures provide the efficient generation of superoxide anions under the action of visible light due to electron transfer from QDs to titania nanoparticles with ~60% efficiency. We also tested the antibacterial activity of hybrid structures on five strains of bacteria. The formed structures combined with visible light irradiation effectively inhibit the growth of *Escherichia coli*, *Bacillus subtilis*, and *Mycobacterium smegmatis* bacteria, the last of which is a photosensitive causative agent model of tuberculosis.

## 1. Introduction

Bacterial infections, despite the high level of development of modern medicine, remain among the most dangerous socially significant diseases in the current era [1], threatening human health, food security, and the development of society in general. The inappropriate use of antibiotics has become one of the reasons for the manifestation of antibiotic resistance in bacteria [2,3]. Many new bacterial strains have been found to be resistant to one or several types of antibiotics every year [4]. That is why in 2017, the World Health Organization (WHO) announced the need to develop new bactericidal systems to combat the growing threat of antimicrobial resistance [5]. In particular, in this statement, the importance of funding the treatment of tuberculosis was noted since multiple strains of tuberculosis pathogens that are resistant to antibiotics, according to WHO statistics, are registered in more than 100 countries around the world. The current situation of the COVID-19 pandemic and the risk of respiratory infections has increased manifold, also indicating the need for new approaches for the treatment of not only bacterial but also viral infections, including respiratory infections, which can significantly complicate the course of coronavirus infection. These facts determine the urgent need to search for new approaches and alternative systems for effective therapy for bacterial infections.

Many pathogenic bacteria can spread with airborne droplets or can settle on various surfaces. This increases the risk of infection through touching objects, walls, and furniture, followed by touching mucous membranes. The creation of bactericidal self-cleaning coatings can solve this problem and can significantly reduce the risk of infection in places where a large number of potential carriers of infection accumulate. Such coatings that are capable of preventing the accumulation of pathogenic bacteria have long been included in the sphere of interest of modern biomedicine [6,7,8]. In the last decade, nanostructured materials have become the focus of attention of researchers who are involved in the development of bactericidal systems that can solve the problem of bacterial resistance to antibiotics [9,10]. It is well known that some nanoparticles are capable of generating reactive oxygen species (ROS) when they are in an excited state when interacting with the surrounding oxygen compounds [11,12], i.e., singlet oxygen [13], superoxide [14], hydroxyl radical [15], peroxide [16], etc. It is well known that ROS are permanently generated as products of normal cellular oxygen metabolism in the mitochondria of living cells and serve as the mediators of intracellular reactions [17,18]. At the same time, an increase in the ROS concentration typically leads to so-called oxidative stress [19,20], resulting in the DNA fragmentation of the cell wall and the penetration of ROS into the cell, causing further damage to the cell organelles, resulting in cell death. This bactericidal mechanism is the basis for the functioning of a large number of bactericidal systems that are based on various nanoparticles [21,22]. Metal oxide nanoparticles (TiO_2_, ZnO, CuO, Ag_2_O, etc.) that are capable of efficient generation of various ROS [23] are of particular interest. Titania (TiO_2_) and ZnO are the undisputed leaders among metal oxide particles for the treatment of bacterial infections [24,25,26,27,28].

A significant disadvantage of ZnO and TiO_2_ is their wide bandgap (3.2 eV for anatase TiO_2_ and 3.3 eV for ZnO) [29]. Therefore, UV radiation (<380 nm) is needed to bring a nanoparticle to an excited state, while UV itself manifests sterilizing properties and is unsafe for the human body [30]. This explains the need for a solution to expand the spectral activity range of particles. One of the approaches to solving this problem is to combine NPs with a semiconductor that has a narrower bandgap, such as A_2_B_6_, with specific examples being cadmium selenide and quantum dots [31]. Due to the mutual arrangement of the conduction bands of QDs with titania NPs, effective photo-induced electron transfer (ET) from QDs to NPs can be realized in hybrid nanostructures that comprise them [32,33,34]. Thus, titania NP/QD hybrid structures combine the optical properties of QDs with the ability of NPs to efficiently generate ROS, resulting in bactericidal activity under visible radiation.

The concept of the sensitization of titania nanoparticle film is attractive since the material is readily available and known for its antimicrobial action; however, it requires additional activation in order to be efficient under visible light. Employing CdSe QDs for activation seems to be an elegant way to create new types of materials. The unique properties of such hybrid structures have led to numerous studies focusing on the bactericidal activity of structures with an original architecture [35,36,37,38]. A few of these studies are devoted to the mechanism of bactericidal activity; in the rest, practical applications for inhibiting bacterial growth are investigated. In addition, there is a lack of work exploring the simplest double structures that could become the optimal model object for research. Unfortunately, the effectiveness of the bactericidal structures that have been developed by different groups can hardly be compared because of a lack of data on their photophysical properties, particularly data regarding the charge transfer efficiency. Moreover, there have been no systematic studies on the bactericidal effects of hybrid structures on a wide range of Gram-positive and Gram-negative bacterial cells with different cell wall thicknesses. In the overwhelming majority of studies, the object for research is *Escherichia coli* [39]. Despite a large number of relevant publications, it currently remains unclear as to whether titania NP/QD hybrid structures can find real-life applications as bactericidal coatings. Obviously, in order to answer this question, the photophysical properties of titania NP/QD hybrid structures and their impact on various bacterial cells need to be examined in detail.

Therefore, we present the results that we obtained during the preparation of titania NP/CdSe QD hybrid structures and during the study of their luminescent properties in order to determine an estimation of the ET efficiency in the structures. Then, we discuss the ROS generation efficiency of those structures using a chemical sensor and present our original approach for the evaluation of ET efficiency from the acceptor side, i.e., using the ROS generated by the titania NPs in the structures. Next, we discuss the results that were obtained and examine the impact of our structures on five bacterial strains, i.e., *Escherichia coli*, *Staphylococcus aureus*, *Bacillus subtilis*, *Pseudomonas aeruginosa*, and *Mycobacterium smegmatis*. Finally, we demonstrate the synergetic effect of our structures with visible light on *Mycobacterium smegmatis*, which is a model of tuberculosis bacteria [40].

## 2. Materials and Methods

### 2.1. Synthesis of Titania NPs and CdSe QDs

Cadmium oxide (Sigma Aldrich, St. Louis, MO, USA), selenium powder (Sigma Aldrich, St. Louis, MO, USA), 2-ethylhexanoic acid (Sigma Aldrich, St. Louis, MO, USA), octadacene (Sigma Aldrich, St. Louis MO USA), oleylamine (Sigma Aldrich, St. Louis, MO, USA), and hexadecylphosphonic acid (Sigma Aldrich, St. Louis, MO, USA), trioctylphosphine (Fluka, Gothenburg, Sweden) were used for the synthesis of colloidal CdSe QDs [41]. For a uniform distribution of QDs during the formation of the hybrid structures, the stabilizer on the QD surface was replaced by oleic acid molecules. For this, an excess of oleic acid (10% of QDs solution volume) was added to the QD solution, and the resulting mixture was left for 48 h at room temperature, after which the excess stabilizer molecules were removed from the sample. See Figure S1 in the Supplementary Materials for their absorbance and luminescence spectra. To synthesize the TiO_2_ nanoparticles, titanium (IV) chloride (Sigma Aldrich, St. Louis, MO, USA), benzyl alcohol (Sigma Aldrich, St. Louis, MOm USA), and oleic acid (Fluka, Gothenburg, Sweden) were used [42]. Hexane (Vekton, Saint-Petersburg, Russia) and isopropyl alcohol (Lenreaktiv, Saint-Petersburg Russia) were used to remove the excess stabilizer molecules from the QDs and from the surface of the titania NPs.

### 2.2. Formation of Titania NP/CdSe QD Multilayered Hybrid Structures

We formed our multilayered hybrid structures using the modified Langmuir–Blodgett technique [43]. Briefly, colloidal solutions of titania nanoparticles and CdSe QDs in toluene were dropped onto the water surface, and titania NP or CdSe QD layers were formed on the water–air interface due to toluene evaporation; then, the layers were transferred to the dielectric substrates (See Section S2 and Figure S2 of the Supplementary Materials for details). Hybrid structures containing ten double layers, each of which comprised a monolayer of CdSe QDs and a monolayer of titania NPs, were formed. See the Supplementary Materials for a detailed characterization of the samples (Section S3: Figures S3–S6). This a hybrid structure architecture was optimal to obtain a correct assessment of the antibacterial activity. A pronounced antibacterial effect was observed within a short period of time, during which other factors could not affect the survival of the tested bacteria.

### 2.3. Study of ROS Generation by the Hybrid Structures

ROS generation was investigated using a selective chemical sensor, *p*-Nitrosodimethylaniline (RNO). During its interaction with ROS, the optical density in the 440 nm absorption band of the sensor decreased due to the one-electron oxidation of its chromophore group [44]. Multilayered hybrid structures were formed on the walls of a dismountable spectroscopic cell (See Figure S7 of the Supplementary Materials), which was filled up with the sensor solution (~10^−5^ M) to study the ROS generation. A mercury lamp (365 nm, 2 mW) and a 465 nm LED (6 mW) were used as sources of external radiation. The UV-3600 Probe spectrophotometer (Shimadzu, Kyoto, Japan) was used for to measure the absorption spectra of the RNO sensor the during external radiation experiments.

### 2.4. Experiments with Bacteria

**Figure 1:**
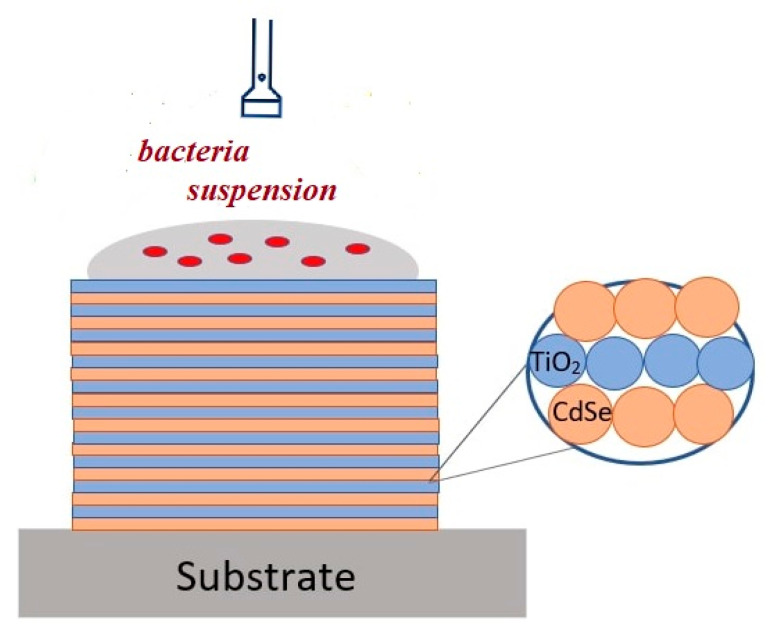
Sketch representation of experiment with bacteria.

Modified agar Gause medium #2, which comprises 1% glucose, 0.5% peptone, 0.3% tryptone, 0.5% NaCl, 2% agar, and tap water with pH 7.2–7.4, was used to store and maintain all of the microorganisms. The same medium without agar was used to cultivate any submerged bacteria. Bacteria were introduced into the liquid medium in the amount of 10^5^ and 2.8 × 10^7^ colony forming units (CFU/mL). The 25 × 10 mm microscope slides with the titania NP/CdSe QD hybrid structures were sterilized by autoclaving in a 0.5 excess atmosphere mode for 30 min. The effect of autoclaving on the luminescence properties and ROS generation efficiency by hybrid structures is discussed in detail in Section S5 of the Supplementary Materials (Figures S8 and S9). Sterile microscope slides were transferred to 35 mm plastic Petri dishes (Eppendorf, Hamburg, Germany). An amount of 75 μL of the bacterial suspensions was applied to microscope slides and to the bottom of an empty Petri dish (reference) (Figure S10 of the Supplementary Materials). Treated Petri dishes were paired together (experiment and the reference) and were placed under a separate LED with a radiation wavelength of 465 nm at a distance of 7 cm from the sample, where is the light power was 3 mW (incident radiation dose = power × exposure time) (Figure S11 of the the Supplementary Materials). Several diodes were used in the experiments, the power of which was the same and constant in time (during these experiments, this was monitored using the PM100USB Power sensor, Thorlabs, Newton, NJ, USA). In the case of daylight, the light intensity was 150 lx (ATE-1509, Aktakom, Moscow, Russia), and the total radiation dose was an order of magnitude lower than that of LED irradiation. The scheme of the experiment is shown in Figure 1.

The exposure time was varied from 2 to 7 h in different experiments. During the experiment, the possible temperature changes that were caused due to the irradiation of the samples were monitored; during prolonged irradiation, the temperature increased by no more than 0.5 °C. After 7 h, no visible changes in the droplet volume of the bacteria suspension were observed (control over the drop area). After irradiation, 10 μL from each drop was plated onto an agar medium in Petri dishes with a diameter of 90 mm without preliminary dilution as well as with dilutions that were greater by 2 and 4 orders of magnitude. The dishes were placed in a MIR-H263-PE thermostat (Sanyo Electric Co., Osaka, Japan) with a temperature of 37 °C. Bacterial colony growth was analyzed every second day for all of the bacteria types except *Mycobacterium smegmatis*, whose growth was assessed after 4–5 days. The antibacterial activity of the structures was calculated relative to the corresponding control, which was taken as 100% bacterial survival. Table 1 provides basic information on the bacterial strains that were used in the experiment.

A hydrophobic coating that contained none of the components of the hybrid structures was used as a reference. All of the bacterial strains were grown in the medium and were placed on a hydrophobic surface, i.e., the bottom of the Petri dishes, and were used in dark, daylight, and 465 nm LED radiation experiments as reference samples. All of the experiments were conducted at room temperature.

## 3. Results and Discussions

The formation of hybrid structures based on CdSe QD will expand the spectral range of titanium dioxide activity [45]. It is considered that cadmium-containing QDs are quite toxic [46,47]. In this situation, it should be noted that the toxicity only manifests itself when the QDs are destroyed and when the Cd ions interact with organism directly [48]. In addition, the developed coatings would not be injected into the body. These factors make it possible to remove the question of the QD toxicity during the formation of hybrid structures, and the experimental results of the work also demonstrate the absence of toxicity.

Titania NP/CdSe QD hybrid structures should efficiently generate ROS under visible radiation within the QD absorption range because of efficient the ET that results from the QDs to the titania nanoparticles [32]. The ET is an additional electronic excitation relaxation channel in the QDs in the hybrid structures that competes with their photoluminescence. Therefore, efficient ET from the QD conduction band should lead to a significant quenching of the QD luminescence and, at the same time, increase the efficiency of ROS generation by the titania NPs. Recently, it has been demonstrated that small-sized CdSe QDs, i.e., 2.5 nm diameter core, are better electron donors for titania NPs than larger ones [49]. It has been also shown that the ZnS shell [50] in core/shell CdSe/ZnS QDs acts as an energy barrier for electron transfer, thus impairing the functionality of hybrid structures. Recently, we have demonstrated [51] (see also the Supplementary Materials, Section S7: Figure S12 and Table S1) that 2.5 nm CdSe QDs are the best ET donors for titania NPs-based nanostructures.

### 3.1. Photophysical Properties of Multilayered Titania NP/CdSe QD Hybrid Structures

**Figure 2:**
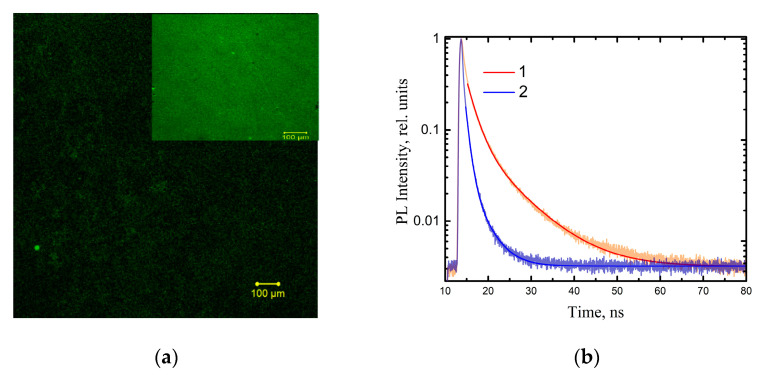
(**a**) PL image of CdSe QD layer before (the insert) and after titania NPs deposition. Area is 1250 × 1250 μm^2^, lens is 10×/0.45, and PL excitation wavelength is 405 nm. (**b**) PL decay of QD layer (1) and Titania NP/QD hybrid structures (2).

Multilayered titania NP/CdSe QD hybrid structures are alternating layers of spherical titania nanoparticles and CdSe QDs (see Section 2 for details). We have found that the deposition of a layer of titania nanoparticles does not significantly affect the morphology of the QDs in the structures. At the same time, the decrease in the QD photoluminescence intensity upon the deposition of a layer of titania nanoparticles is clearly observed in Figure 2.

As seen in the PL images (Figure 2a), the deposition of titania nanoparticles leads to a significant quenching of the QD luminescence compared to the QD layers (Figure 2a Insert). The formation of titania NP/CdSe QD hybrid structures should be accompanied by the efficient quenching of QDs luminescence due to the photo-induced electron transfer from the QDs to the titania NPs [32,33,34]. It is possible to estimate the electron transfer efficiency based on the assumption that it is the only channel where PL quenching is observed. The estimates were conducted on the basis of QD PL kinetics according to method that we developed in an earlier study [52]. Our calculations showed that the formation of hybrid structures is accompanied QD luminescence of up to 80% (see Section S8, Figure S13 and Table S2 in the Supplementary Materials for details).

Obviously, any effective bactericidal system has to generate ROS under external radiation and within a broad spectral range, including visible light, which is safe for the human body. The ROS concentration that generated by any bactericidal system and that is based on titania NPs or other metal oxide NPs is univocally related to the number of electrons in the metal oxide conduction band [53]:

(1) $$N_{ROS}=N_{e}\times E_{ROS}$$

where *N_e_* is the number of electrons in the conduction band of the titania nanoparticles as a result of UV radiation absorption; *E_ROS_* is the ROS generation efficiency by the titania NPs.

The efficiency of ROS generation by the hybrid structures can be evaluated by determining the photobleaching of the chemical sensor *p*-nitrosodimethylaniline (RNO) at the wavelength of 440 nm [44] (see Section 2 for the details). It should be noted that EPR and ESR are often used to study the efficiency of ROS generation [54,55]. However, these methods are also based on the chemical conversion of substances and are not characterized by greater reliability compared to more widely used chemical sensors. The change in the optical density of the sensor is proportional to the concentration of the generated ROS:

(2) $$C_{ROS}∼\Delta D_{RNO}$$

The number of electrons generated in a titania nanoparticle per unit time at direct UV radiation absorption can be estimated as:

(3) $$N_{e}^{direct}=(1-T_{\lambda }^{TiO_{2}})\times W_{\lambda }^{direct}$$

where $\left(1-T_{\lambda }^{TiO_{2}}\right)$ is the portion of external radiation that is absorbed by the titania nanoparticles that is proportional to their extinction coefficient (*ε*), and $W_{\lambda }^{direct}$ is the number of photons with energy hc/λ incident on a nanoparticle layer per unit time.

**Figure 3:**
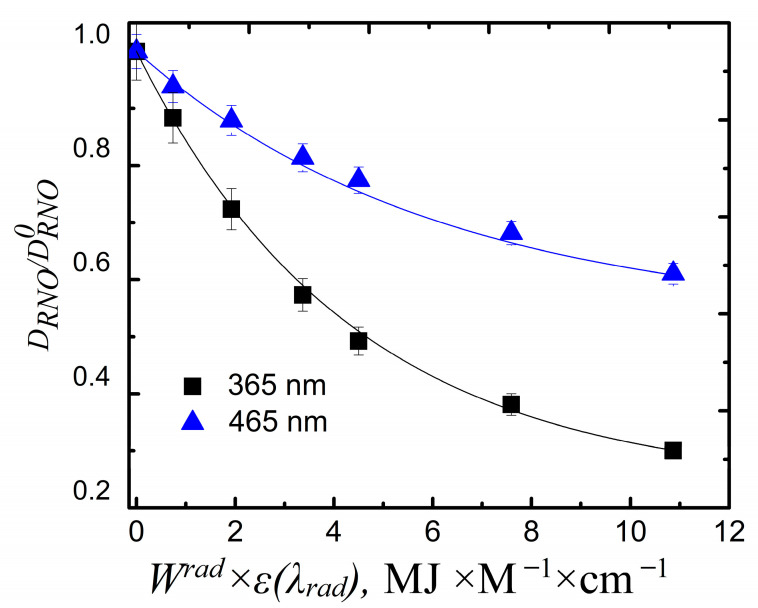
Comparison of ROS generation by titania NPs under exposure to UV (365 nm) and titania NP/CdSe QD hybrid structures under exposure to visible light (465 nm): the extent of sensor bleaching vs. the product of incident energy and the extinction coefficient at the corresponding wavelength (*W^rad^ × ε(λrad*) characterizes the radiation dose absorbed by hybrid structures).

The sensor bleaching that takes place during the course of the exposure of the titania NP/CdSe QD hybrid structures to visible radiation and the exposure of the titania NPs to UV light is compared in Figure 3, where the normalized optical density of the sensor at 440 nm is plotted versus the product of the incident light energy and the extinction coefficient at the wavelength of irradiation, which was 365 or 465 nm, thus characterizing the absorbed irradiation dose.

We demonstrated that our structures were able to efficiently generate ROS under visible light, as seen in Figure 3. Superoxide anion production is the most plausible in titania NP-based structures [56]. We suggest that the ET resulting from the QDs to the titania NPs in our structures is one of the reasons for superoxide anion production because ROS generation by neither of the structure components separately was observed under 465 nm LED irradiation (see Figure S14 of the Supporting Information). This permits the conclusion that the structure functionality depends on (i) the photocatalytic activity of titania NPs and (ii) the efficiency of ET from the QDs to the titania NPs. The latter determines the concentration of ROS under visible radiation:

(4) $$N_{ROS}\sim N_{e}^{sens}=N_{e}^{QDs}\times E_{ET}=\left(1-T_{\lambda }^{QDs}\right)\times W_{\lambda }^{sens}\times E_{ET}$$

where $N_{e}^{QDs}$ is the number of electrons generated in the QD layer under the influence of incident radiation; $E_{ET}$ is the efficiency of electron transfer from a QD to a titania nanoparticle; $\left(1-T_{\lambda }^{QDs}\right)$ is the fraction of incident radiation absorbed by the QD layer; and $W_{\lambda }^{sens}$ is the number of photons with energy hc/λ incident on the QD layer per unit time.

The average ET efficiency was estimated as 55% in our structures when using the method that we described in our previous publication [57]. Our results clearly demonstrate (see Section S5 of Supporting for details) that the effectiveness of ROS generation by the hybrid structures is very well correlated with the 80% PL quenching that was observed in the QDs in the structures. The discrepancy between the ET efficiency that was evaluated from both the acceptor and donor points, i.e., titania NPs and CdSe QDs, respectively, because of the so-called “dark” fraction in the CdSe QD ensemble [58], which was characterized by a high rate of nonradiative decay. 

Our hybrid structures exhibit efficient ROS generation under visible light due to the efficient ET from the CdSe to the titania NPs and can be recommended as a key component for bactericidal systems and coatings. However, the study of the photophysical properties of the hybrid structures is only insufficient for the assessment of their potential effectiveness in the treatment of bacterial infections. Therefore, the next necessary step in creating an effective therapeutic system is the study of the interaction of the hybrid structures with various bacterial strains.

### 3.2. Interaction of Titania NP/CdSe QD Multilayered Hybrid Structures with Bacteria

The bactericidal efficacy of multilayered hybrid structures was studied using five different bacteria strains, including both Gram-positive and Gram-negative bacteria. The experiment is detailed in Section 2.4. Table 2 presents that data that were obtained to determine the viability of the bacteria that were grown on the structures and that were irradiated with 465 nm LED (the total dose of the incident energy was ~75 J).

The Titania NP/CdSe QD hybrid structures demonstrated high bactericidal activity in three out of the five bacteria strains that were studied, namely *E. coli* ATCC 25922, *B. subtilis* ATCC 6633, and *M. smegmatis* mc^2^ 155, as seen in Table 2. It is well known that superoxide anions, similar to other anions, are unable to penetrate the cell [59]. Therefore, we suppose that the observed bactericidal effect is due to the destruction of the bacterial cell wall or another structural difference as well as other electrochemical interactions at the cell wall level [60]. Gram-positive and Gram-negative bacteria differ in the composition of the cell wall [61]. Gram-positive bacteria are characterized by a greater cell wall thickness [62]: in Gram-negative bacteria, it is about 10 nm vs. 20–50 nm in Gram-positive bacteria. According to numerous studies, Gram-positive bacteria are less susceptible to bactericidal systems [60]. This fact makes it possible to assume that Gram-positive bacteria should demonstrate lower bactericidal activity in the titania NP/CdSe QD structures. Our structures showed maximum bactericidal activity against Gram-positive *B. subtilis ATCC 6633* (99.9%). At the same time, our results suggest that *E. coli*, which is used as a standard in the study of bactericidal activity, only shows a 40% bactericidal effect, which is the least pronounced positive effect in our study. This fact can be explained by the presence of the particular superoxide dismutase enzyme that is produced in the *E. coli* cell wall and that reduces the effects of oxidative stress [63]. It should be noted that in many studies determining the bactericidal activity of *E. coli*, this enzyme is often specifically removed [64]. This enzyme is also present in the envelope of *M. smegmatis* [65]. The superoxide dismutase enzyme concentration is the lowest in *B. subtilis* [66], and it can explain why our structures show the highest effect against this type of bacteria. Therefore, we can conclude that the concentration of the superoxide dismutase enzyme in bacteria with thin cell walls may be a key factor that inhibits the bactericidal effect of our hybrid structures.

**Figure 4:**
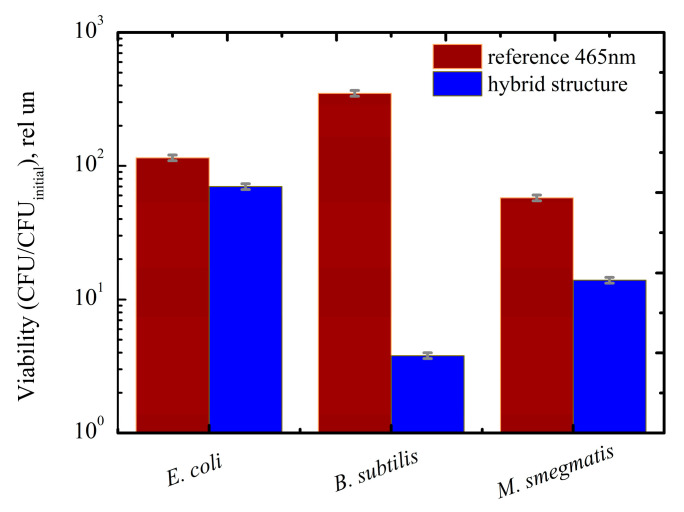
Viability of bacteria grown on titania NP/CdSe QD hybrid structures exposed to 465 nm LED (75 J). Viability was estimated relative to initial CFU of bacteria, CFU_initial_.

The viability of the bacteria when the titania NP/CdSe QD hybrid structures were under the 465 nm irradiation is presented in Figure 4 in comparison with the reference (see Section 2 for details).

We found that the bactericidal activity of our structures is especially pronounced in *B. subtilis* bacteria because of low rate of dismutase enzyme production in the cell wall [67]. It is noteworthy that the *B. subtilis* bacterium is also characterized by the highest titer increase in the reference samples (see Figure 4). This confirms that the photo-induced bactericidal activity of our structures has no relationship with the viability of a particular bacterial strain under visible light and is solely due to the generation of ROS. Despite these facts, *B. subtilis* bacterium is beyond the focus of our interest because it is not pathogenic for the human body.

**Figure 5:**
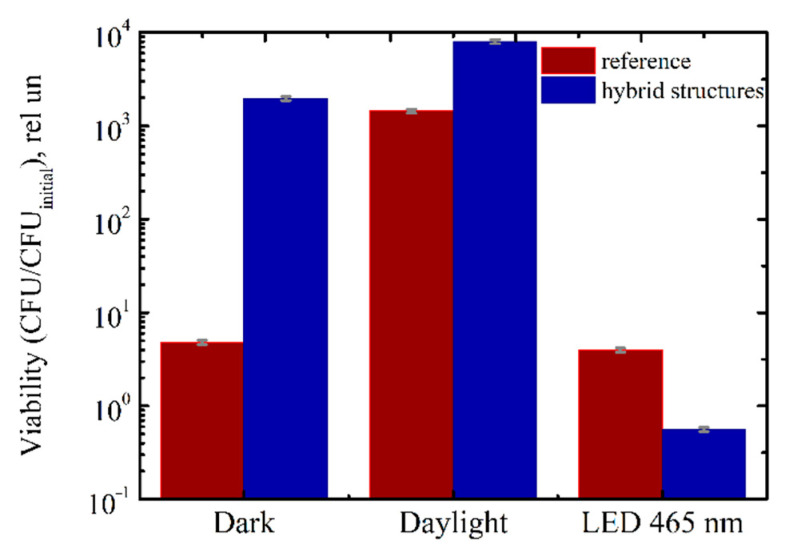
Viability of *Mycobacterium smegmatis* mc^2^ 155 bacteria in the dark, at daylight, and under 465 nm LED exposure (~43 J). Viability was estimated relative to the initial CFU of bacteria, CFU_initial_.

We suppose that *M. smegmatis* mc^2^ 155 is of the greatest interest among the bacteria for which our structures demonstrate the most pronounced bactericidal effect. This bacterium is a model object for tuberculosis and is of particular interest for research in the context of a coronavirus infection pandemic. This bacterium is sensitive to visible light [40]. It explains the poorest growth of this bacterium under 465 nm LED irradiation, see Figure 5. Therefore, it is of particular interest to evaluate the impact of the combination of visible light and our hybrid structures on the *M. smegmatis* bacterium.

The strong impact of lighting conditions on *M. smegmatis* viability in our samples and in the references is shown in Figure 4.

It was surprising that the *M. smegmatis* mc^2^ 155 bacteria grew in our samples, i.e., on the surface of our hybrid structures, more efficiently in the dark and under daylight than in the reference samples, see Figure 5. We suppose that this peculiar feature is due to a difference in terms of the hydrophobization of our samples and those from the references. The energy of daylight incident on the sample is too low (~150 lx) for any noticeable superoxide generation by the hybrid structures. The higher dark and daylight viability of the bacteria that was observed in the hybrid structures strongly confirms the absence of dark toxicity in the structures due to the chemical composition or the topology of their surface as well as the surface absorption of the bacterial cells [68]. In addition, this also confirms the absence of QD toxicity due to the presence of Cd [49]. Figure S15 and Table S3 of the Supplementary Materials provides additional details and results from these experiments.

We observed (see Figure 5) that the 465 nm LED irradiation of the reference samples caused a slight inhibition in the bacteria growth (17% decrease) due to the photosensitivity of *M. smegmatis* mc^2^ 155. By contrast, the visible light irradiation of the *M. smegmatis* mc^2^ 155 bacteria on the surface of our hybrid structures strongly affects their viability, as evidenced by an 86% decrease compared to the reference samples (see Figure 4).

The fact there is an absence of a bactericidal effect in the hybrid structure in the dark and under daylight illumination unambiguously indicates that the ROS generation that resulted from the photo-induced ET from the CdSe QDs to the titania NPs is the main reason for the synergistic effect of the 465 nm LED irradiation and our materials.

**Figure 6:**
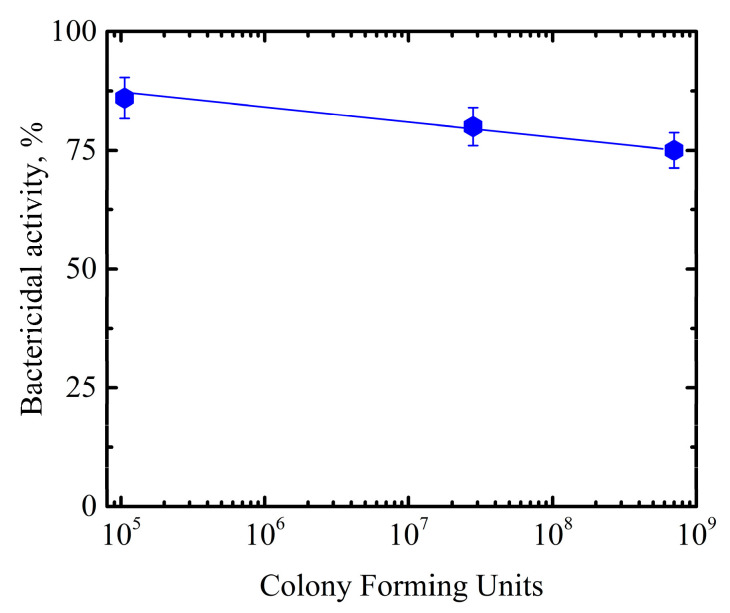
Bactericidal activity of the hybrid structures as a function of the initial *M. smegmatis* mc^2^ 155 suspension titer.

We also found that the bactericidal activity of our hybrid structures only weakly depends on the number of colony-forming units in the titer of the initial *M. smegmatis* mc^2^ 155 bacteria suspension, as seen in Figure 6, where the activity is well expressed within a wide range of the initial titer.

Our results show a slight decrease in the bactericidal activity of the hybrid structures: from 85% to 76%, as the number of bacteria in the initial suspension increases by three orders of magnitude. On average, over the entire titer range, the bactericidal activity of the titania NP/CdSe QD hybrid structures is about 80%.

## 4. Conclusions

We have demonstrated that titania NP/CdSe QD hybrid structures can be used as a base for bactericidal coatings in order to provide efficient protection from airborne infections such as tuberculosis in public places. This effect can be achieved due to the combination of visible light and the hybrid structures that were developed here because of efficient electron transfer from the CdSe QDs to the titania NPs in our structures. We suggest that the superoxide anions efficiently destroy bacterial cell walls, thus leading to the death of bacteria with a high concentration of superoxide dismutase.

Our results clearly demonstrate the high potential of the titania NP/CdSe QD hybrid structures in bactericidal coatings and should exhibit a pronounced effect, even against light-sensitive bacteria and respiratory tract pathogens, which is especially important because of their high resistance to antibiotics and strong interference with SARS-CoV.

## Figures and Tables

**Table 1:** Bacteria strains.

Bacteria *	Type
*Escherichia coli* ATCC 25922	Gram-negative
*Pseudomonas aeruginosa* ATCC 27853	Gram-negative
*Staphylococcus aureus* FDA 209P	Gram-positive
*Bacillus subtilis* ATCC 6633	Gram-positive
*Mycobacterium smegmatis* mc^2^ 155	Gram-positive

* The cell and CFU titers of *Bacillus subtilis* and *Pseudomonas aeruginosa* are equal. For the other three bacteria strains, the growth point corresponds not to a single colony, but to a group of cells.

**Table 2:** Viability of five bacterial strains grown on the structures and irradiated with 465 nm LED (75 J of incident energy).

	*E. coli* *ATCC 25922*, CFU/mL	*P. aeruginosa* *ATCC 27853*, CFU/mL	*S. aureus* *FDA 209P*, CFU/mL	*B. subtilis* *ATCC 6633*, CFU/mL	*M. smegmatis* mc^2^ 155, CFU/mL
Reference samples *	1.2 × 10^7^	~2.4 × 10^9^	~6 × 10^6^	3.5 × 10^7^	5.8 × 10^6^
Titania NP/CdSe QD hybrid structures	7 × 10^6^	~2.4 × 10^9^	~10^7^	3.8 × 10^5^	~1.5 × 10^6^
Decrease in the bacteria number by a factor	~1.5	1	1	~90	~4

* for a description of reference samples, see Methods.

## Data Availability

The data presented in this study are available upon request from the corresponding author.

## Supplementary Materials

The following are available online at https://www.mdpi.com/article/10.3390/nano11123331/s1, Figure S1: Absorption and photoluminescence spectra of CdSe QDs in hexane. The PL excitation wavelength is 405 nm, Figure S2: Photos of a home-made bath used to form hybrid structures by the modified Langmuir-Blodgett method, Figure S3: Scanning electron microscope image of CdSe QDs with size distribution, Figure S4: Scanning electron microscope image of TiO2 NPs layers, Figure S5: Scanning electron microscope image of CdSe QDs layer, Figure S6: AFM image (a) and the height profile (b) of CdSe/TiO2 hybrid structures, Figure S7: Dismountable cell (a) and the cell window with hybrid structures formed on it (b), Figure S8: PL decay of Titania NPs/QD hybrid structures. The smooth lines are results of biexponential fitting with $y=A_{1}\exp(-\frac{t}{\tau_{1}})+A_{2}\exp(-\frac{t}{\tau_{2}})$, Figure S9: Normalized optical density in the absorption band of RNO (D_RNO_/D^0^_RNO_, where D_RNO_ is optical density of the sensor at 440 nm, D^0^_RNO_ is initial optical density) as a function of the irradiation time for hybrid structures before (1) and after (2) autoclaving. The lines are guides for the eye, Figure S10: Images of a suspension of M smegmatis inoculated on multilayer hybrid structures (right) and the bottom of a Petri dish (used as a reference), Figure S11: Images of a suspension of M smegmatis inoculated on multilayer hybrid structures (right) and the bottom of a Petri dish (used as a reference), Figure S12: Normalized optical density in the absorption band of RNO (*D*_RNO_/*D*^0^_RNO_, where *D*_RNO_ is optical density of the sensor at 440 nm, *D*^0^_RNO_ is initial optical density) as a function of the absorbed radiation dose characterized by the product of extinction coefficient and incident doze, for three types of hybrid structures. The lines are guides for the eye., Figure S13: PL decay of dry QD layers (1) and Titania NPs/QD hybrid structures. (2). The smooth lines are results of biexponential fitting with $y=A_{1}\exp(-\frac{t}{\tau_{1}})+A_{2}\exp(-\frac{t}{\tau_{2}})$, Figure S14: Normalized optical density in the absorption band of RNO (*D*_RNO_/*D*^0^_RNO_, where *D*_RNO_ is optical density of the sensor at 440 nm, *D*^0^_RNO_ is initial optical density) as a function of the external radiation dose (465 nm) for Titania NPs (1) and CdSe QDs (2). The lines are guides for the eye, Figure S15: *M smegmatis* growth in the reference samples at different conditions of external irradiation, Table S1: Dependence of the electron transfer efficiency on the structure architecture for three types of QDs, Table S2: Fit-parameters, i.e. PL decay time (*τ_i_*) and PL amplitude (*A_i_*), of biexponential fitting of PL kinetics of QDs in a dry layer and in the hybrid structures, Table S3: Viability of *M. smegmatis* mc^2^ 155 bacteria at different lighting conditions.

## References

[1] Shiadeh M.N., Moghadam Z.B., Adam I., Saber V., Bagheri M., Rostami A. (2017). Human infectious diseases and risk of preeclampsia: An updated review of the literature. Infection.

[2] Zaman S.B., Hussain M.A., Nye R., Mehta V., Mamun K.T., Hossain N. (2017). A review on antibiotic resistance: Alarm bells are ringing. Cureus.

[3] Ben Y., Fu C., Hu M., Liu L., Wong M.H., Zheng C. (2019). Human health risk assessment of antibiotic resistance associated with antibiotic residues in the environment: A review. Environ. Res..

[4] Butler M.S., Blaskovich M.A., Cooper M.A. (2017). Antibiotics in the clinical pipeline at the end of 2015. J. Antibiot..

[5] World Health Organization (WHO) Global Priority List of Antibiotic-Resistant Bacteria to Guide Research, Discovery, and Development of New Antibiotics. http://www.who.int/medicines/publications/WHO-PPL-Short_Summary_25Feb-ET_NM_WHO.pdf?ua=1.

[6] Montali A. (2006). Antibacterial coating systems. Injury.

[7] Benetti G., Cavaliere E., Brescia R., Salassi S., Ferrando R., Vantomme A., Van Bael M.J. (2019). Tailored Ag–Cu–Mg multielemental nanoparticles for wide-spectrum antibacterial coating. Nanoscale.

[8] Nosrati R., Olad A., Shakoori S. (2017). Preparation of an antibacterial, hydrophilic and photocatalytically active polyacrylic coating using TiO_2_ nanoparticles sensitized by graphene oxide. Mater. Sci. Eng. C.

[9] Allafchian A., Hosseini S.S. (2019). Antibacterial magnetic nanoparticles for therapeutics: A review. IET Nanobiotechnol..

[10] Shrestha A., Kishen A. (2016). Antibacterial nanoparticles in endodontics: A review. J. Endod..

[11] Latvala S., Hedberg J., Di Bucchianico S., Möller L., Odnevall Wallinder I., Elihn K., Karlsson H.L. (2016). Nickel release, ROS generation and toxicity of Ni and NiO micro-and nanoparticles. PLoS ONE.

[12] Abdal Dayem A., Hossain M.K., Lee S.B., Kim K., Saha S.K., Yang G.M., Cho S.G. (2017). The role of reactive oxygen species (ROS) in the biological activities of metallic nanoparticles. Int. J. Mol. Sci..

[13] Qin Y., Chen L.J., Dong F., Jiang S.T., Yin G.Q., Li X., Yang H.B. (2019). Light-controlled generation of singlet oxygen within a discrete dual-stage metallacycle for cancer therapy. J. Am. Chem. Soc..

[14] Azócar M.I., Alarcón R., Castillo A., Blamey J.M., Walter M., Paez M. (2019). Capping of silver nanoparticles by anti-inflammatory ligands: Antibacterial activity and superoxide anion generation. J. Photochem. Photobiol. B Biol..

[15] Li N., Liu X. (2019). Synthesis of dendrimer-stabilized au nanoparticles and their application in the generation of hydroxyl radicals. ChemistrySelect.

[16] Ran P., Song J., Mo F., Wu J., Liu P., Fu Y. (2019). Nitrogen-doped graphene quantum dots coated with gold nanoparticles for electrochemiluminescent glucose detection using enzymatically generated hydrogen peroxide as a quencher. Microchim. Acta.

[17] Shi K., Gao Z., Shi T.Q., Song P., Ren L.J., Huang H., Ji X.J. (2017). Reactive oxygen species-mediated cellular stress response and lipid accumulation in oleaginous microorganisms: The state of the art and future perspectives. Front. Microbiol..

[18] Angelova P.R., Abramov A.Y. (2016). Functional role of mitochondrial reactive oxygen species in physiology. Free Radic. Biol. Med..

[19] Fetoni A.R., Paciello F., Rolesi R., Paludetti G., Troiani D. (2019). Targeting dysregulation of redox homeostasis in noise-induced hearing loss: Oxidative stress and ROS signaling. Free Radic. Biol. Med..

[20] Farooq M.A., Niazi A.K., Akhtar J., Farooq M., Souri Z., Karimi N., Rengel Z. (2019). Acquiring control: The evolution of ROS-Induced oxidative stress and redox signaling pathways in plant stress responses. Plant Physiol. Biochem..

[21] Raghunath A., Perumal E. (2017). Metal oxide nanoparticles as antimicrobial agents: A promise for the future. Int. J. Antimicrob. Agents.

[22] Hemeg H.A. (2017). Nanomaterials for alternative antibacterial therapy. Int. J. Nanomed..

[23] Li Y., Zhang W., Niu J., Chen Y. (2012). Mechanism of photogenerated reactive oxygen species and correlation with the antibacterial properties of engineered metal-oxide nanoparticles. ACS Nano.

[24] Ivask A., Kurvet I., Kasemets K., Blinova I., Aruoja V., Suppi S., Visnapuu M. (2014). Size-dependent toxicity of silver nanoparticles to bacteria, yeast, algae, crustaceans and mammalian cells in vitro. PLoS ONE.

[25] Sirelkhatim A., Mahmud S., Seeni A., Kaus N.H.M., Ann L.C., Bakhori S.K.M., Mohamad D. (2015). Review on zinc oxide nanoparticles: Antibacterial activity and toxicity mechanism. Micro Nano Lett..

[26] Parasuraman P., Antony A.P., Sharan A., Siddhardha B., Kasinathan K., Bahkali N.A., Syed A. (2019). Antimicrobial photodynamic activity of toluidine blue encapsulated in mesoporous silica nanoparticles against Pseudomonas aeruginosa and Staphylococcus aureus. Biofouling.

[27] Cano A., Ettcheto M., Espina M., López-Machado A., Cajal Y., Rabanal F., Souto E.B. (2020). State-of-the-art polymeric nanoparticles as promising therapeutic tools against human bacterial infections. J. Nanobiotechnol..

[28] Rauf A., Sarwar H.S., Amin U., Naveed S., Ali I., Razzaq S., Shahnaz G. (2020). Tuberculosis resistance and nanoparticles: Combating the dual role of reactive oxygen species in macrophages for tuberculosis management. Crit. Rev. Ther. Drug Carrier Syst..

[29] Pekárek S., Mikeš J., Krýsa J. (2015). Comparative study of TiO_2_ and ZnO photocatalysts for the enhancement of ozone generation by surface dielectric barrier discharge in air. Appl. Catal A Gen..

[30] Benson R.S. (2002). Use of radiation in biomaterials science. Nucl. Instrum. Methods Phys. Res. B Beam Interact. Mater. Atoms.

[31] Liu Y., Zhou H., Zhou B., Li J., Chen H., Wang J., Cai W. (2011). Highly stable CdS-modified short TiO_2_ nanotube array electrode for efficient visible-light hydrogen generation. Int. J. Hydrogen Energy.

[32] Jin S., Lian T. (2009). Electron transfer dynamics from single CdSe/ZnS quantum dots to TiO_2_ nanoparticles. Nano Lett..

[33] Lin K.H., Chuang C.Y., Lee Y.Y., Li F.C., Chang Y.M., Liu I.P., Lee Y.L. (2012). Charge transfer in the heterointerfaces of CdS/CdSe cosensitized TiO_2_ photoelectrode. J. Phys. Chem. C.

[34] Wang L., Han J., Feng J., Wang X., Su D., Hou X., Dou S.X. (2019). Simultaneously efficient light absorption and charge transport of CdS/TiO_2_ nanotube array toward improved photoelectrochemical performance. Int. J. Hydrogen Energy.

[35] Ma X., Xiang Q., Liao Y., Wen T., Zhang H. (2018). Visible-light-driven CdSe quantum dots/graphene/TiO_2_ nanosheets composite with excellent photocatalytic activity for *E. coli* disinfection and organic pollutant degradation. Appl. Surf. Sci..

[36] Gao P., Liu J., Zhang T., Sun D.D., Ng W. (2012). Hierarchical TiO_2_/CdS “spindle-like” composite with high photodegradation and antibacterial capability under visible light irradiation. J. Hazard. Mater..

[37] Kang Q., Lu Q.Z., Liu S.H., Yang L.X., Wen L.F., Luo S.L., Cai Q.Y. (2010). A ternary hybrid CdS/Pt–TiO_2_ nanotube structure for photoelectrocatalytic bactericidal effects on *Escherichia coli*. Biomaterials.

[38] Lu Z.X., Zhang Z.L., Zhang M.X., Xie H.Y., Tian Z.Q., Chen P., Pang D.W. (2005). Core/shell quantum-dot-photosensitized nano-TiO_2_ films: Fabrication and application to the damage of cells and DNA. J. Phys. Chem B.

[39] Palmer B.R., Marinus M.G. (1994). The dam and dcm strains of *Escherichia coli*—A review. Gene.

[40] Ragusa J., Gonzalez D., Li S., Noriega S., Skotak M., Larsen G. (2019). Glucosamine/L-lactide copolymers as potential carriers for the development of a sustained rifampicin release system using Mycobacterium smegmatis as a tuberculosis model. Heliyon.

[41] Sukhanova A., Even-Desrumeaux K., Chames P., Baty D., Artemyev M., Oleinikov V., Nabiev I. (2012). Engineering of ultra-small diagnostic nanoprobes through oriented conjugation of single-domain antibodies and quantum dots. Protoc. Exch..

[42] Niederberger M., Bartl M.H., Stucky G.D. (2002). Benzyl alcohol and transition metal chlorides as a versatile reaction system for the nonaqueous and low-temperature synthesis of crystalline nano-objects with controlled dimensionality. J. Am. Chem. Soc..

[43] Alaferdov A.V., Savu R., Rackauskas S., Rackauskas T., Canesqui M.A., Gromova Y.A., Moshkalev S.A. (2015). New Hybrid Structures Based on CdSe/ZnS Quantum Dots and Multilayer Graphene for Photonics Applications.

[44] Muff J., Bennedsen L.R., Søgaard E.G. Detailed parameter study on the mechanisms in electrochemical oxidation of p-nitrosodimethylaniline in chloride electrolyte. Proceedings of the 2nd European Conference on Environmental Applications of Advanced Oxidation Processes.

[45] Etacheri V., Di Valentin C., Schneider J., Bahnemann D., Pillai S.C. (2015). Visible-light activation of TiO_2_ photocatalysts: Advances in theory and experiments. J. Photochem. Photobiol. C: Photochem. Rev..

[46] Moulis J.M., Thévenod F. (2010). New perspectives in cadmium toxicity: An introduction. Biometals.

[47] Yong K.T., Law W.C., Hu R., Ye L., Liu L., Swihart M.T., Prasad P.N. (2013). Nanotoxicity assessment of quantum dots: From cellular to primate studies. Chem. Soc. Rev..

[48] Li K.G., Chen J.T., Bai S.S., Wen X., Song S.Y., Yu Q., Wang Y.Q. (2009). Intracellular oxidative stress and cadmium ions release induce cytotoxicity of unmodified cadmium sulfide quantum dots. Toxicol. In Vitro.

[49] Robel I., Kuno M., Kamat P.V. (2007). Size-dependent electron injection from excited CdSe quantum dots into TiO_2_ nanoparticles. J. Am. Chem. Soc..

[50] Zhu H., Song N., Lian T. (2010). Controlling charge separation and recombination rates in CdSe/ZnS type I core−shell quantum dots by shell thicknesses. J. Am. Chem. Soc..

[51] Kolesova E.P., Safin F.M., Maslov V.G., Dubavik A., Gun’ko Y.K., Orlova A.O. (2020). Photophysics of Titania Nanoparticle/Quantum dot hybrid structures. Opt. Spectrosc..

[52] Kolesova E.P., Safin F.M., Maslov V.G., Gun’ko Y.K., Orlova A.O. (2019). The influence of photoinduced processes on a Quantum dot surface on the electron transfer efficiency in TiO_2_ Nanoparticle/Quantum dot structures. Opt. Spectrosc..

[53] Kolesova E., Maslov V., Safin F., Purcell-Milton F., Cleary O., Volkov Y., Gun’ko Y.K., Orlova A. (2019). Photoinduced charge transfer in hybrid structures based on titanium dioxide NPs with multicomponent qd exciton luminescence decay. J. Phys. Chem. C.

[54] Saita M., Kobatashi K., Yoshino F., Hase H., Nonami T., Kimoto K., Masaichi C.I. (2012). ESR investigation of ROS generated by H_2_O_2_ bleaching with TiO_2_ coated HAp. Dent. Mater. J..

[55] Suzen S., Gurer-Orhan H., Saso L. (2017). Detection of reactive oxygen and nitrogen species by electron paramagnetic resonance (EPR) technique. Molecules.

[56] Kőrösi L., Bognár B., Bouderias S., Castelli A., Scarpellini A., Pasquale L., Prato M. (2019). Highly-efficient photocatalytic generation of superoxide radicals by phase-pure rutile TiO2 nanoparticles for azo dye removal. Appl. Surf. Sci..

[57] Kolesova E.P., Maslov V.G., Gun’ko Y.K., Orlova A.O. (2019). A Method for estimating the functionality of TiO_2_/Quantum dot multilayer hybrid structures based on the generation of reactive oxygen species. Opt. Spectrosc..

[58] Durisic N., Wiseman P.W., Grütter P., Heyes C.D. (2009). A common mechanism underlies the dark fraction formation and fluorescence blinking of quantum dots. ACS Nano.

[59] Imlay J.A. (2013). The molecular mechanisms and physiological consequences of oxidative stress: Lessons from a model bacterium. Nat. Rev. Microbiol..

[60] Agarwal A., Makker K., Sharma R. (2008). Clinical relevance of oxidative stress in male factor infertility: An update. Am. J. Reprod. Immunol..

[61] Fu L.M., Fu-Liu C.S. (2002). Is Mycobacterium tuberculosis a closer relative to Gram-positive or Gram–negative bacterial pathogens?. Tuberculosis.

[62] Cummins C.S., Harris H. (1956). The chemical composition of the cell wall in some gram-positive bacteria and its possible value as a taxonomic character. Microbiology.

[63] Guzman M., Dille J., Godet S. (2012). Synthesis and antibacterial activity of silver nanoparticles against gram-positive and gram-negative bacteria. Nanomed. Nanotechnol. Biol. Med..

[64] Touati D., Jacques M., Tardat B., Bouchard L., Despied S. (1995). Lethal oxidative damage and mutagenesis are generated by iron in delta fur mutants of *Escherichia coli*: Protective role of superoxide dismutase. J. Bacteriol. Res..

[65] Kusunose E., Ichihara K., Noda Y., Kusunose M. (1976). Superoxide dismutase from Mycobacterium tuberculosis. J. Biochem..

[66] Gregory E.M., Fridovich I. (1973). Oxygen toxicity and the superoxide dismutase. J. Bacteriol. Res..

[67] Bertrand R.L., Eze M.O. (2013). *Escherichia coli* superoxide dismutase expression does not change in response to iron challenge during lag phase: Is the ferric uptake regulator to blame?. Adv. Enzyme Res..

[68] Tripathy A., Sen P., Su B., Briscoe W.H. (2017). Natural and bioinspired nanostructured bactericidal surfaces. Adv. Colloid Interface Sci..

