# Supplementary material for: Bactericidal Activity of Multilayered Hybrid Structures Comprising Titania Nanoparticles and CdSe Quantum Dots under Visible Light
Source: Nanomaterials (Basel). 2021 Dec 8;11(12):3331. doi: 10.3390/nano11123331 (PMC8708662; doi:10.3390/nano11123331)
Supplement: Supplementary file 1 [file nanomaterials-11-03331-s001.zip › nanomaterials-1454904-supplementary.pdf]

nanomaterials

Supplementary Materials

Bactericidal Activity of Multilayered Hybrid Structures Comprising Titania Nanoparticles and CdSe Quantum Dots under Visible Light

Ekaterina Kolesova [1,*], Anastasia Bulgakova [1], Vladimir Maslov [1], Andrei Veniaminov [1], Aliaksei Dubavik [1], Yurii Gun'ko [2], Olga Efremenkova [1,3], Vladimir Oleinikov [1,4] and Anna Orlova [1,*]

[1] School of Photonics, ITMO University, 197101 St. Petersburg, Russia; anastasiya.makovectkaya@mail.ru (A.B.); maslov04@bk.ru (V.M.); avveniaminov@itmo.ru (A.V.); adubavik@yandex.ru (A.D.); ovefr@yandex.ru (O.E.); voleinik@mail.ru (V.O.)
[2] School of Chemistry, Trinity College Dublin, D02 PN40 Dublin, Ireland; igounko@tcd.ie
[3] Department of Microbiology, FSBI Gause Institute of New Antibiotics, 119021 Moscow, Russia
[4] Department of Biomaterials and Bionanotechnology, Shemyakin-Ovchinnikov Institute of Bioorganic Chemistry, 117997 Moscow, Russia
* Correspondence: e.p.kolesova@gmail.com (E.K.); a.o.orlova@gmail.com (A.O.)

S1. Optical Properties of CdSe QDs

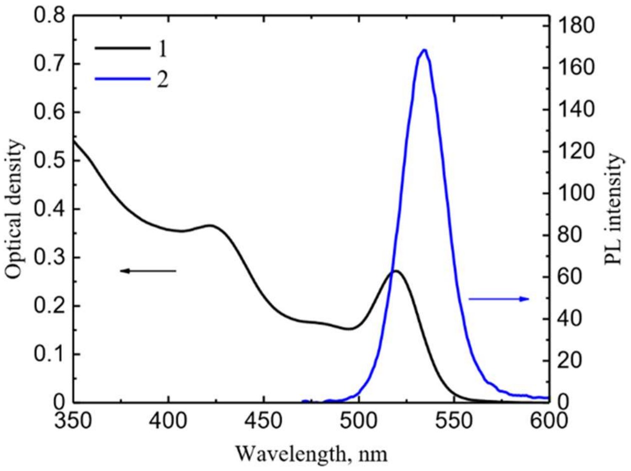

Figure S1. Absorption (1) and photoluminescence (2) spectra of CdSe QDs in hexane. The PL excitation wavelength is 405 nm.

S2. Formation of Hybrid Structures

We have applied the modified Langmuir-Blodgett method to form our hybrid structures using a home-made trough presented in Figure S3. Briefly, 15 μL of QDs (5×10^{-6} M) or Titania NPs (2×10^{-6} M) hexane solutions were dropped onto deionized water (conductivity 18 MΩ·cm). Then, hexane was evaporating for 30 minutes, resulting in the NPs distributed on the water surface. At the final stage, the thus prepared NPs films were transferred from water to a glass slide. The glass slide was tilted and fixed at the 45° angle to the water surface and then water was slowly drained from the trough.

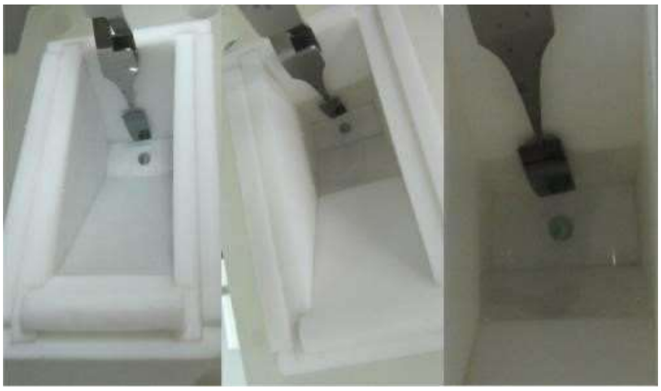

Figure S2. Photos of a home-made bath used to form hybrid structures by the modified Langmuir-Blodgett method.

S3. Multilayered Titania NPs/CdSe Structures

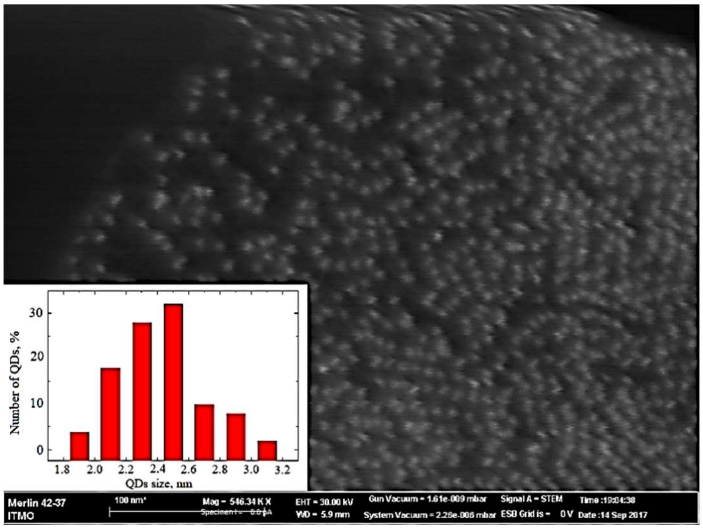

Figure S3. Scanning electron microscope image of CdSe QDs with size distribution (inset).

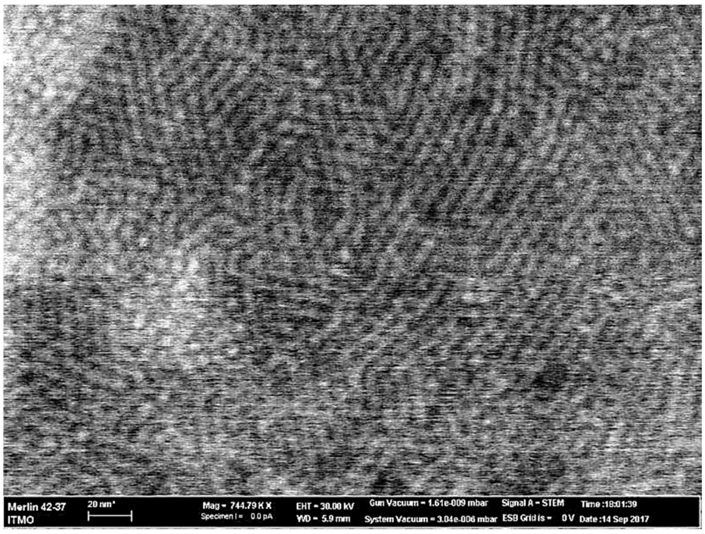

Figure S4. Scanning electron microscope image of TiO_2 NPs layers.

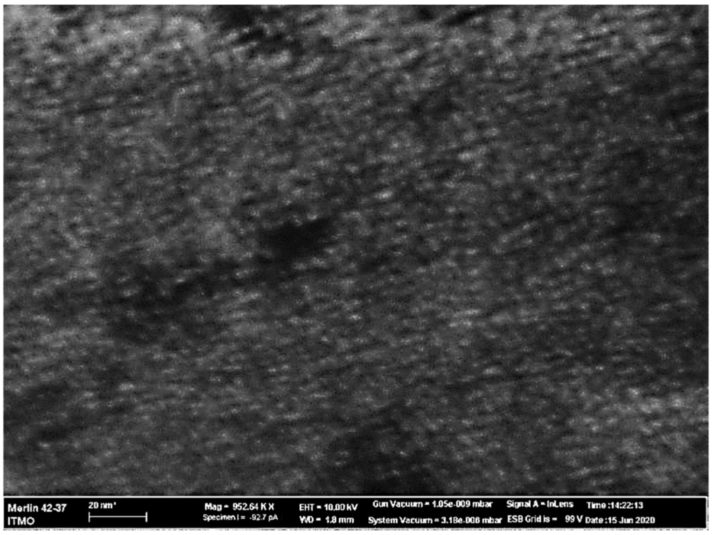

Figure S5. Scanning electron microscope image of CdSe QDs layer.

Figure S6 shows AFM image and the height profile of TiO_2/QDs hybrid structures formed by modified Langmuir-Blodgett technique and containing 10 layers of components.

a b

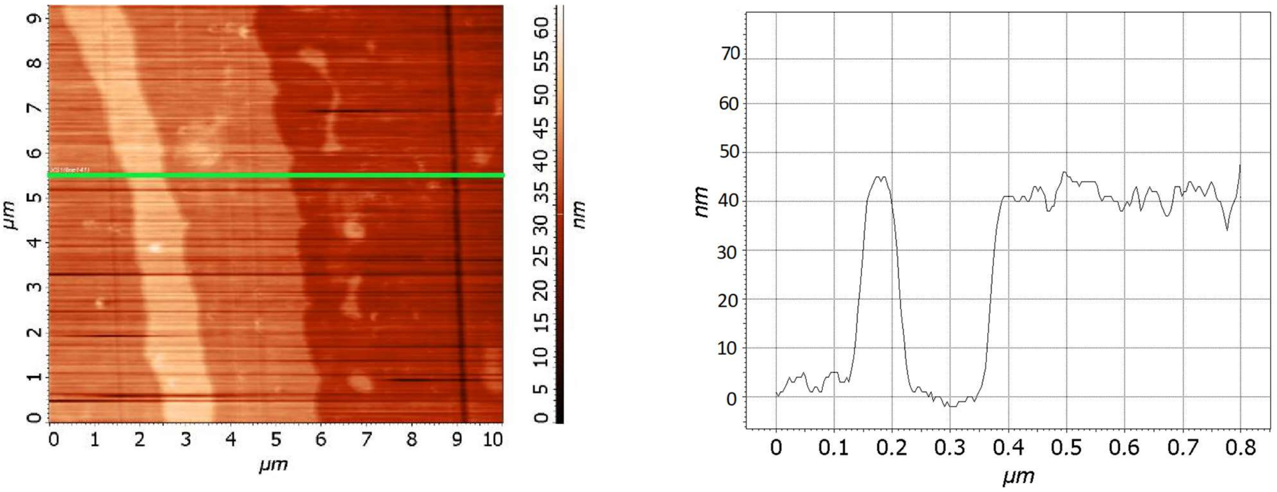

Figure S6. AFM image (**a**) and the height profile (b) of CdSe/TiO_2 hybrid structures.

S4. Investigation of ROS Generation

Figure S7(a) shows the spectroscopic cell used for ROS generation studies with a chemical sensor. Briefly, hybrid structures, QDs and Titania NPs were layered onto the cell windows presented in Figure S7(b) using the modified Langmuir-Blodgett method. Then the cell was assembled and filled with the sensor solution.

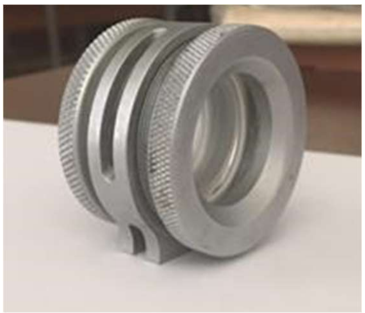

(**a**)

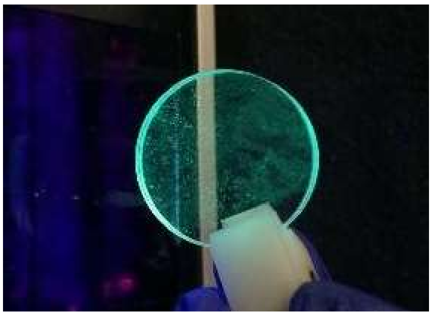

(**b**)

Figure S7. Dismountable cell (**a**) and the cell window with hybrid structures formed on it (**b**).

S5. Effect of Autoclaving on the Photophysical Properties of Titania Nanoparticles/CdSe Structures

The luminescence properties and the ability of titania NPs/CdSe structures to generate ROS under visible radiation after autoclaving of the structures were analyzed. Figure S8 shows PL decay curve of titania NPs/CdSe structures.

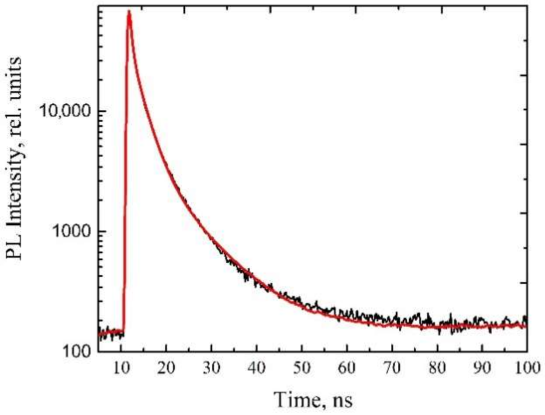

Figure S8. PL decay of Titania NPs/QD hybrid structures. The smooth lines are results of biexponential fitting with $y = A_1 \exp(-\frac{t}{\tau_1}) + A_2 \exp(-\frac{t}{\tau_2})$.

The fit parameters coincide with the data before autoclaving, it can be concluded that autoclaving does not affect the luminescent properties of QDs in the structures.

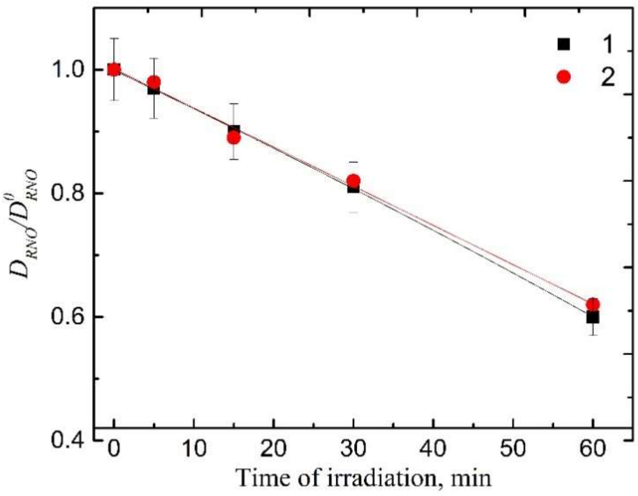

Figure S9. Normalized optical density in the absorption band of RNO (D_{RNO}/D^0_{RNO}, where D_{RNO} is optical density of the sensor at 440 nm, D^0_{RNO} is initial optical density) as a function of the irradiation time for hybrid structures before (1) and after (2) autoclaving. The lines are guides for the eye.

S6. Sample Preparation for Bactericidal Activity Studies

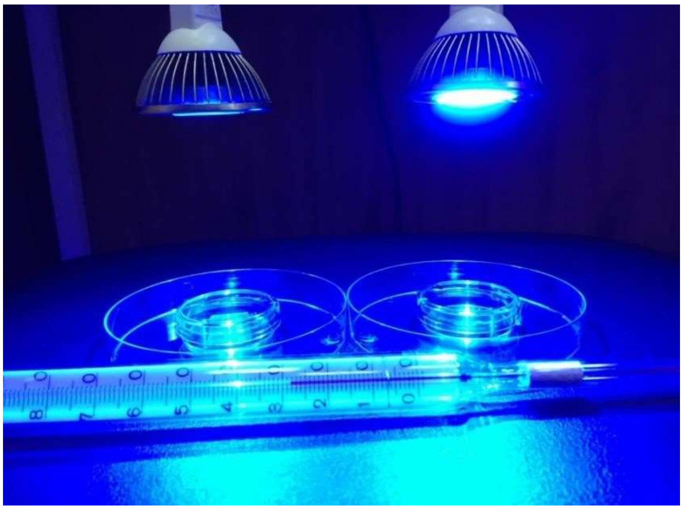

Figure S10. A suspension of *M smegmatis* mc^2 *155* seeded on multilayer hybrid structures and irradiated with 465 nm LED.

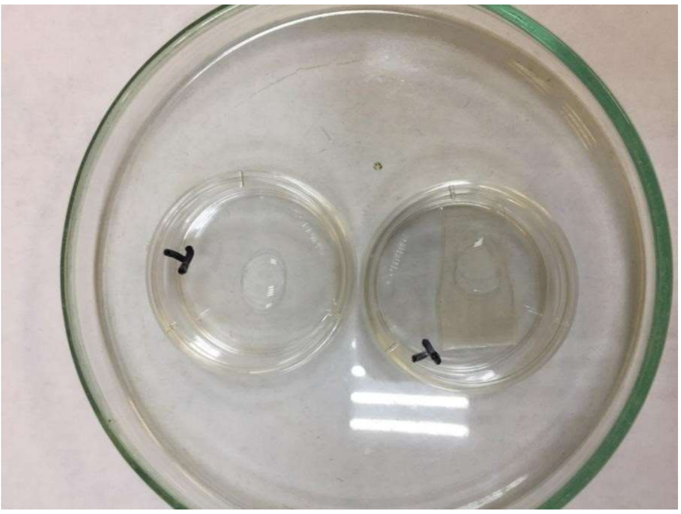

Figure S11. Images of a suspension of *M smegmatis* inoculated on multilayer hybrid structures (right) and the bottom of a Petri dish (used as a reference).

S7. Investigation of ROS Generation by Hybrid Structures Based on Different Types of QDs.

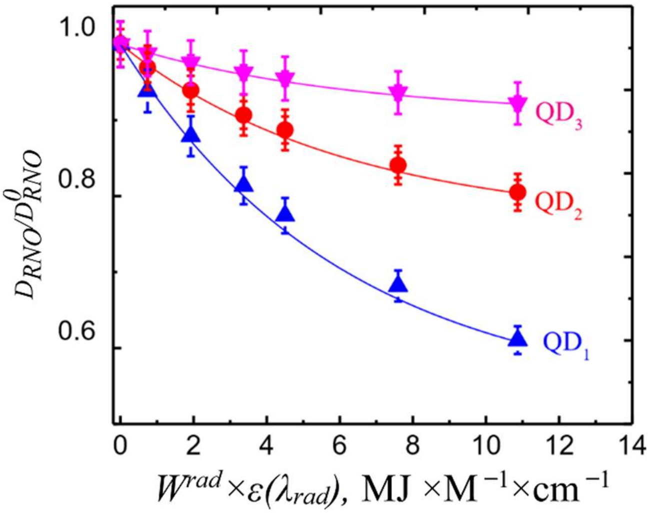

Figure S12. Normalized optical density in the absorption band of RNO (D_{RNO}/D^0_{RNO}, where D_{RNO} is optical density of the sensor at 440 nm, D^0_{RNO} is initial optical density) as a function of the absorbed radiation dose characterized by the product of extinction coefficient and incident doze ($W^{rad}\times\varepsilon(\lambda rad)$), for three types of hybrid structures. The lines are guides for the eye.

Table S1. Dependence of the electron transfer efficiency on the structure architecture for three types of QDs.

	Type of QDs	Architecture	Electron Transfer Efficiency, %
QD_1	CdSe Core	2.5 nm core	60 ± 5
QD_2	CdSe Core/ ZnS Shell	5.5 nm core, 0.5 nm shell	27 ± 2
QD_3	CdSe Core/ ZnS Shell	2.5 nm core, 4.5 nm shell	9 ± 1

S8. Evaluation of CdSe QDs Luminescence Quenching

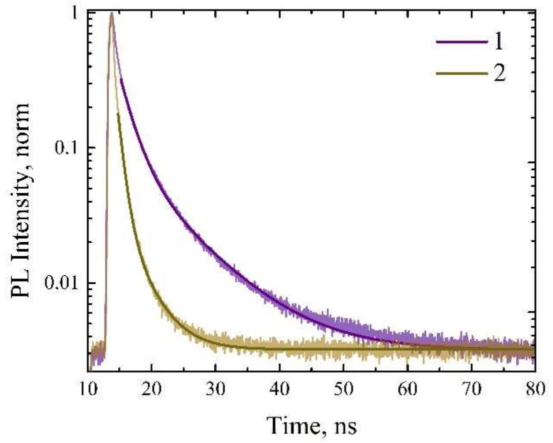

Figure S13. PL decay of dry QD layers (1) and Titania NPs/QD hybrid structures. (2). The smooth lines are results of biexponential fitting with $y = A_1 \exp(-\frac{t}{\tau_1}) + A_2 \exp(-\frac{t}{\tau_2})$.

Table S2. Fit-parameters, i.e. PL decay time (τ_i) and PL amplitude (A_i), of biexponential fitting of PL kinetics of QDs in a dry layer and in the hybrid structures.

	τ_1, ns	A_1, rel. units	τ_2, ns	A_2, rel. units
CdSe QD	8 ± 1	3300 ± 300	2.0 ± 0.2	10,500 ± 1000
TiO_2 NP/CdSe QD	3.0 ± 0.5	1500 ± 150	1.0 ± 0.1	5600 ± 500

The deposition of TiO_2 nanoparticles on the QD layer leads to a reduction in both the characteristic PL decay times and in their amplitudes, as can be seen from Table S2. Under given conditions for recording the PL decay curves, the decrease in the amplitudes A_i indicates a decrease in the QDs number in the fraction with a particular decay time. A decrease in the PL amplitudes allows concluding that PL of some QDs in these fractions was totally quenched.

The efficiency of the luminescence quenching for QD ensemble can be estimated using the following formula:

$$Q = 1 - \frac{I}{I0} \quad \text{(S1)}$$

where I and I_0 are PL intensity values in the presence and the absence of a quencher, respectively.

To analyze the PL quenching efficiency using eq. S1, it is necessary to estimate the change in the PL intensity during the formation of the hybrid structures. All luminescent QD fractions make additive contributions to the PL intensity of the QD ensemble. The PL intensity of the *i-th* subensemble of QDs is proportional to the QD concentration and the quantum yield of their PL:

$$I^i \sim C^i \times \varphi^i \sim A^i \times \frac{\tau^i}{\tau_r} \quad \text{(S2)}$$

where C^i is the concentration, A^i is the amplitude at $t = 0$, proportional to the concentration, φ^i is PL quantum yield, τ^i is PL decay time, τ_r is radiative decay time, for *i*-th subensemble. It is assumed that all QD subensembles have identical absorption spectra.

Using Equations (S1)–(S2), PL quenching can be estimated as follows:

$$Q = 1 - \frac{I}{I_0} = 1 - \frac{\sum_{i=1}^{k} A^i \times \frac{\tau^i}{\tau_r}}{\sum_{i=1}^{k} A_0^i \times \frac{\tau_0^i}{\tau_r}} \quad \text{(S3)}$$

The quenching of QD luminescence during the formation of hybrid structures was estimated as 80% using Equation (S3). Under the assumption that all the PL quenching of QDs in the hybrid structures is due to electron transfer from QD to TiO_2 nanoparticle, the obtained value corresponds to the efficiency of electron transfer in the hybrid structures

S9. ROS Generation by Titania NPs and CdSe QDs under Visible Light

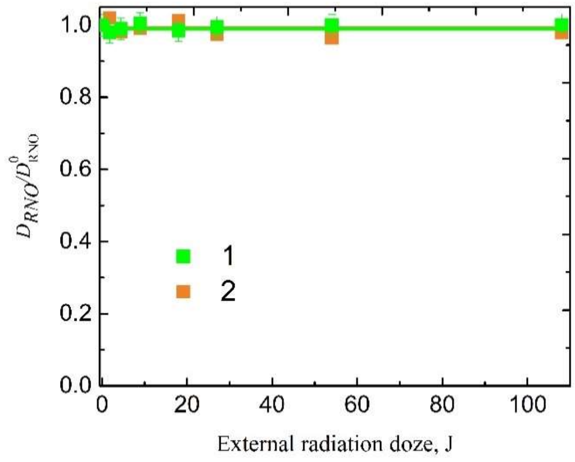

Figure S14. Normalized optical density in the absorption band of RNO (D_{RNO}/D^0_{RNO}, where D_{RNO} is optical density of the sensor at 440 nm, D^0_{RNO} is initial optical density) as a function of the external radiation dose (465 nm) for Titania NPs (1) and CdSe QDs (2). The lines are guides for the eye.

Irradiation of hybrid structures' components, Titania NPs and CdSe QDs, does not lead to bleaching of the sensor when the samples are irradiated with visible light. This clearly indicates the absence of superoxide anion generation under 465 nm LED.

S10. Viability of Bacterial Strains under Irradiation of Hybrid Structures

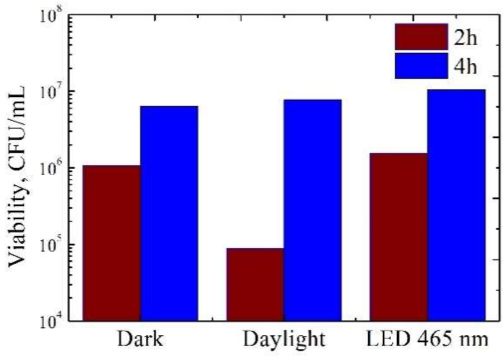

Figure S15. *M smegmatis* growth in the reference samples at different conditions of external irradiation.

Table S3. Viability of *M. smegmatis* mc[2] 155 bacteria at different lighting conditions.

**Samples**	**465 nm LED**	**Daylight**	**Dark**
Titania NPs/CdSe QDs hybrid structures	5.6×10^3	8.0×10^7	2.0×10^7
Reference samples *	4.0×10^4	1.4×10^7	4.8×10^4

* See Materials and Methods section for details.
